# CHK Methylation Is Elevated in Colon Cancer Cells and Contributes to the Oncogenic Properties

**DOI:** 10.3389/fcell.2021.708038

**Published:** 2021-06-29

**Authors:** Shudong Zhu, Yan Zhu, Qiuwen Wang, Yi Zhang, Xialing Guo

**Affiliations:** ^1^School of Medicine, Nantong University, Nantong, China; ^2^Department of Biochemistry and Molecular Biology, School of Life Science, Central South University, Changsha, China; ^3^Xuzhou Health Research Institute, Xuzhou, China; ^4^Department of Gastrointestinal Surgery, The Third Xiangya Hospital, School of Life Science, Central South University, Changsha, China; ^5^Argus Pharmaceuticals, Changsha, China

**Keywords:** CHK, colon cancer, DNA methylation, FHC, drug resistance

## Abstract

Src is an important oncogene that plays key roles in multiple signal transduction pathways. Csk-homologous kinase (CHK) is a kinase whose molecular roles are largely uncharacterized. We previously reported expression of CHK in normal human colon cells, and decreased levels of CHK protein in colon cancer cells leads to the activation of Src (Zhu et al., 2008). However, how CHK protein expression is downregulated in colon cancer cells has been unknown. We report herein that CHK mRNA was decreased in colon cancer cells as compared to normal colon cells, and similarly in human tissues of normal colon and colon cancer. Increased levels of DNA methylation at promotor CpG islands of CHK gene were observed in colon cancer cells and human colon cancer tissues as compared to their normal healthy counterparts. Increased levels of DNA methyltransferases (DNMTs) were also observed in colon cancer cells and tissues. DNA methylation and decreased expression of CHK mRNA were inhibited by DNMT inhibitor 5-Aza-CdR. Cell proliferation, colony growth, wound healing, and Matrigel invasion were all decreased in the presence of 5-Aza-CdR. These results suggest that increased levels of DNA methylation, possibly induced by enhanced levels of DNMT, leads to decreased expression of CHK mRNA and CHK protein, promoting increased oncogenic properties in colon cancer cells.

## Introduction

Src is an important oncogene that plays key roles in multiple signal transduction pathways (Yeatman, 2004). Csk-homologous kinase (CHK) sharing 53% amino acid identity with c-Src tyrosine kinase (Csk). CHK has been reported to be expressed primarily in brain and hematopoietic cells (Chow et al., 1994). Unlike Csk, which phosphorylates and inhibits Src effectively, CHK is not capable of phosphorylating Src Y530 effectively (Advani et al., 2017). The molecular and functional roles of CHK are largely uncharacterized.

We previously reported that CHK expression was not restricted to brain and hematopoietic cells, instead, CHK is also expressed in normal colon cells and its protein levels were decreased in colon cancer cells, leading to the activation of Src *via* a mechanism irrelevant of Src Y530 phosphorylation (Zhu et al., 2008). We also showed that CHK acts additively with Csk in suppressing Src kinase activity in colon cells (Zhu et al., 2008). Recently, it is also shown that CHK plays an auxiliary role to Csk in hematopoietic cells (Nagy et al., 2020).

The main objective of this current study was to explore the mechanisms of how CHK protein levels are downregulated in colon cancer cells. Here, we reported CHK mRNA was also decreased in colon cancer cells as compared to normal colon cells, and decreased in human colon cancer tissues as compared to normal colon tissues. Underlying molecular mechanism was studied. Biological effects were also studied in the presence of 5-Aza-CdR. The results suggest that increased levels of DNA methylation, possibly induced by enhanced levels of DNA methyltransferases (DNMTs), leads to decreased expression of CHK mRNA and therefore CHK protein, promoting increased oncogenic properties, revealing an important CHK regulatory mechanism in contributing to the tumorigenesis of colon cancer.

## Materials and Methods

### Materials

5-Aza-CdR was purchased from Sigma. GAPDH antibody was from Cell Signaling. DNMT3a and DNMT3b antibodies were from Cellclonal, China. DNMT1 antibody was from Wanlei, China.

### Cell Culture

SW48, SW480, RKO, LoVo, HCT-15, HCT 116, HT-29, FHC, and K562 (ATCC) were cultured in the complete growth medium according to ATCC. Primary normal colon epithelial cells were obtained from normal colon tissues and cultured in DMEM/F12 complete medium.

### Preparation of Lysates

Subconfluent cells in tissue culture were lysed in RIPA lysis buffer supplemented with phosphatase inhibitors and protease inhibitors (Zhu et al., 2007). Lysates were clarified by centrifugation at 10,000 *g* for 20 min. Protein was quantified with the BCA assay. Tumor tissues were homogenized in RIPA lysis buffer with a Dounce homogenizer for 50 strokes first before centrifugation (Bjorge et al., 2000).

### Immunoblotting

Proteins were separated on SDS-PAGE followed by transfer to PVDF membranes. Blots were incubated with the primary antibodies in TBST at room temperature for 1 h. Following incubation with secondary antibody conjugated to horseradish peroxidase, the blots were visualized with ECL.

### Quantitative Real-Time PCR

Total RNA from cell or tissue samples were isolated using TRIzol reagent (Invitrogen) and reverse transcription was done using HiScript IIQ RT superMix (Vazyme, China) following instructions of the manufacture. cDNA was amplified using PCR primers as follows: forward 5′- CTGTCCTGCAGGGTGAGTACCT -3′ and reverse 5′- GTCATGACGGCCGTCTCGTCC -3′. RT-qPCR results were analyzed using comparative cycle threshold (Ct), followed by gene expressions normalized to that of GAPDH.

### Methylation-Specific PCR

Total DNA of the cells or tissues was extracted using TIANamp Genomic DNA kit (TianGen Biotech, China) according to instructions of the manufacturer. Bisulfite conversion of the DNA was done using EZ DNA Methylation – Gold Kit (Zymo, United States). Methylation-specific PCR primers were as follows: (Methylation) forward 5′- AGGTGTGCGTATACGTTTTC -3′ and reverse 5′- TATACGCGACCCTACGTAAC -3′. (Unmethylation) forward 5′- AATAGGTGTGTATATGTTTTT -3′ and reverse 5′- TATACACAACCCTACATAACACC -3′. PCR products were examined using agarose gel electrophoresis followed by images captured with Gel Doc XR + (Bio-Rad).

### Methyl-thiazolyldiphenyl-tetrazoliumbromide Assay

Cells were treated with 5-Aza-CdR for various hours as stated in the figure legends. Methyl-thiazolyldiphenyl-tetrazoliumbromide (MTT) assay of Succinate Dehydrogenase (SDH) activity was performed as described (Molenaar et al., 2012).

### Clonogenic Growth Assay

Thousand cells were plated in 6-well plates and cultured for 14 days in presence or absence of 5-Aza-CdR, with medium changed every three days until colonies were evident. The plates were washed with PBS, fixed with paraformaldehyde and stained with hematoxylin. Images were taken by the inverted microscopy. Colonies were counted manually in a blinded fashion.

### Wound Healing Assay

Cells were plated in 6-well plates to a density of about 80–90% confluence. A linear wound was generated with a 200 μl pipette tip straightly. Cell fragments were immediately removed by using PBS and then cells were cultured in medium supplement with 1% FBS. Images were taken at 0, 24, and 48 h of wound formation by the inverted microscopy and the width of the scratch was measured using ImageJ software.

### Cell Invasion Assay

This was done by Matrigel Invasion Chamber (BD Biosciences) and stained with Diff Quik, according to the protocols of the manufacturer.

### Statistical Analysis

The results were expressed as means ± SD. Experiments were repeated for three independent times unless stated otherwise. The difference of two groups was assessed by analysis of variance (ANOVA) and *t* test.

## Results

**FIGURE 1:**
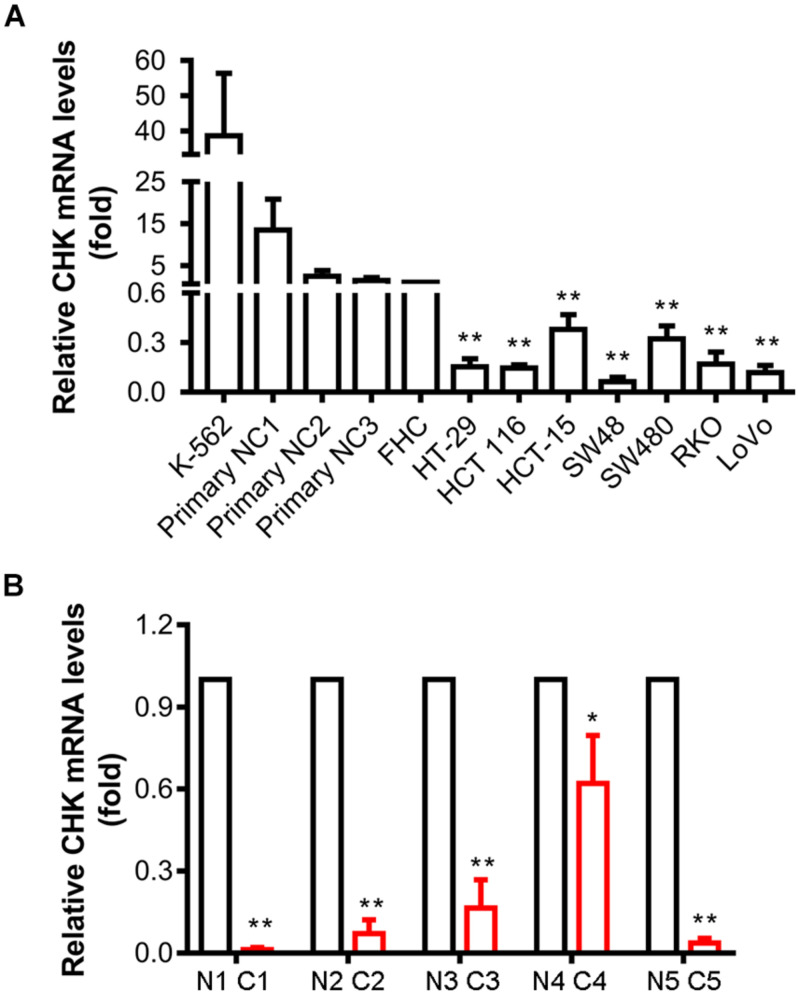
Expression of CHK mRNA is downregulated in human colon cancer cell lines and colon cancer tissues. mRNA levels were measured using qPCR. **(A)** Cell lines. FHC was control normal colon cell strain. K562 was a positive control of CHK mRNA expression. NC, normal colon. **(B)** Tissues. C, cancer tissue; N, adjacent normal tissue. #, patient number. The relative expression level was calculated according to the Ct value. *t*-test, *P < 0.05, **P < 0.01. Error bars represent the SD from three independent experiments.

Downregulation of CHK protein levels activates Src in colon cancer cells (Zhu et al., 2008). To explore if transcriptional regulation led to this downregulation of CHK protein, we examined the mRNA levels of CHK in various colon cell lines using qPCR. Results show that mRNA levels in all the colon cancer cell lines examined, including HT-29, HCT 116, HCT-15, SW48, SW480, RKO, and LoVo, were all significantly decreased as compared to those in normal colon epithelial cell strain FHC (Figure 1A). To further verify the results, we also employed primary colon epithelial cells from human normal colon tissues of three individuals, and found similar downregulation of CHK mRNA as compared to these primary normal colon epithelial cells (Figure 1A).

Tumor tissues and adjacent normal tissues of five colorectal cancer patients have also been collected for examination of the CHK mRNA levels. Levels of CHK mRNA in tumor tissues were significantly lower than those in corresponding normal tissues (Figure 1B).

**FIGURE 2:**
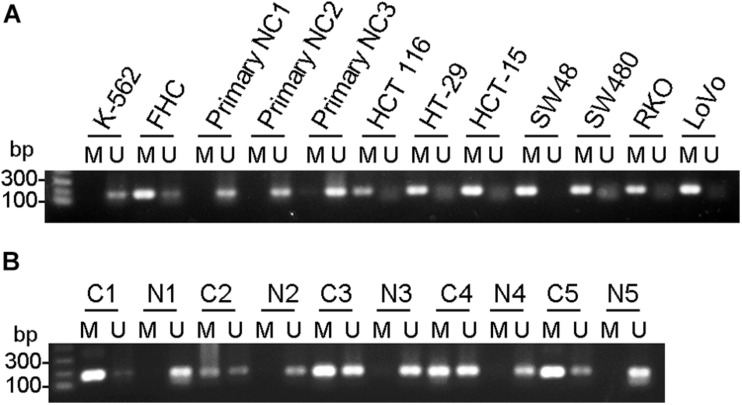
CHK gene was methylated in colon cancer cell lines and colon cancer tissues. Detection of the 196-bp product specific for a bisulfite conversion of genomic DNA was shown. U and M indicate the presence of unmethylated and methylated PCR products, respectively. **(A)** Cells. FHC was control normal colon cell strain. K562 was a positive control of CHK unmethylation. NC, normal colon. **(B)** Tissues. C, cancer tissue; N, adjacent normal tissue. #, patient number. Results are representative of three independent experiments.

To investigate if the mRNA decrease is due to epigenetic regulation, we examined methylation of CpG islands at CHK promoter region. In all colorectal cancer cells examined, including HT-29, HCT 116, HCT-15, SW48, SW480, RKO and LoVo, and CpG islands were heavily methylated, suggesting CHK mRNA were poorly expressed. In contrast, in the three control primary normal colon epithelial cells, methylation of CpG islands were undetectable, suggesting high levels of CHK mRNA expression (Figure 2A). On the other hand, although FHC was methylated at CpG islands, the level of unmethylation is higher than those of the colon cancer cell lines, in line with that the level of CHK mRNA in FHC is higher than those in the colon cancer cell lines.

The change of methylation of CpG islands of the CHK gene were also examined in colorectal cancer tissues and adjacent tissues and showed similar pattern (Figure 2B). In #1 and #5 patients, CHK gene was fully methylated or nearly fully methylated in the cancer tissues, in contrast to full unmethylation or almost full unmethylation in the adjacent normal colon tissues. In #2, #3, and #4 patients, although unmethylation was detected in colon cancer tissues, the percentages of unmethylated CHK in the total CHK in normal colon tissues were much higher than that in colon cancer tissues (Figure 2B).

These data show consistent upregulation of methylation at CHK CpG island in the colon cancer cell lines and cancer tissues.

The down-regulation of CHK mRNA and the upregulation of the CHK CpG methylation suggest that the downregulation of CHK protein in colorectal cancer cells may be caused by downregulation of CHK mRNA, and the downregulation of CHK mRNA in colorectal cancer cells may be caused by the methylation of the CpG island of the CHK gene.

CpG methylation is mediated by DNMTs. We next examined the levels of expression of various DNMTs, including DNMT1, DNMT3a, and DNMT3b. Immunoblotting results indicate that the expression of DNMT1 protein in all the colon cancer cells including HT-29, HCT 116, HCT-15, SW48, SW480, RKO (except LoVo) were all elevated compared with normal colonic epithelial cells including primary cells of normal colon (primary cell #1, 2, and 3), and normal colon epithelial cells FHC (Supplementary Figure 1A). Although DNMT1 level in LoVo cells appear to be similar to that in the FHC and primary normal colon 2, and 3 cells, it is in fact higher than that in these cells after normalization with GAPDH.

On the other hand, The expression of DNMT3a is low in normal colonic epithelial cells FHC and primary normal colon epithelial cells 1, 2, and 3, and relatively higher in colon cancer cells (HT-29, HCT 116, HCT-15, SW48, SW480, RKO, and LoVo). This is also true for DNMT3b (Supplementary Figure 1A). Taken together, the expression levels of DNMTs are higher in colon cancer cells than that in normal colon cells.

Furthermore, the expression of DNMT1 and DNMT3b were all elevated in human colon cancer tissues as compared to the adjacent normal colon tissues (Supplementary Figure 1B). Although the DNMT3a protein levels appears to be elevated in cancer tissues as compared with the adjacent normal colon tissues as well, the image quality of immunoblotting results appears low, probably due to low abundance and degradation of DNMT3a in the tissues (data not shown).

**FIGURE 3:**
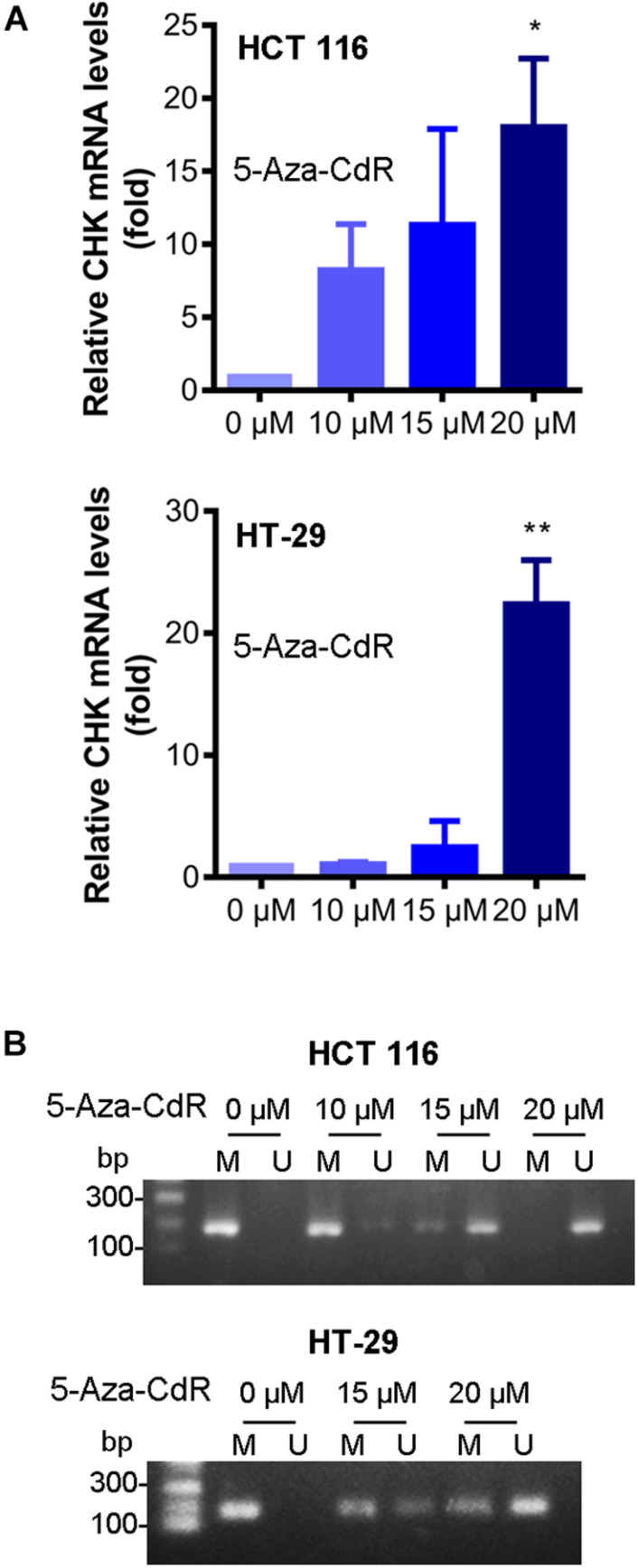
5-Aza-CdR reversed CHK methylation and expression of CHK mRNA. HCT 116 and HT-29 cells were treated with indicated concentrations of 5-Aza-CdR. mRNA levels were measured using qPCR **(A)**. The relative expression level was calculated according to the Ct value. *t*-test, *P < 0.05, **P < 0.01. *N* = 3 independent experiments. Methylation of CHK gene in colon cancer cell lines was measured **(B)**. Detection of the 196-bp product specific for a bisulfite conversion of genomic DNA was shown. U and M indicate the presence of unmethylated and methylated PCR products, respectively. Results are representative of three independent experiments.

The expression of CHK mRNA was increased, after treatment of the cells with DNMT inhibitor 5-Aza-CdR (Figure 3A). Furthermore, the methylation levels of CHK gene were decreased after treatment of the cells with 5-Aza-CdR (Figure 3B). These results are in line with the observed differences of CHK gene methylation and expression of CHK mRNA in between colon cancer cells and normal colon cells, suggesting that DNA methylation plays an important role in silencing CHK mRNA expression in the colon cancer cells.

**FIGURE 4:**
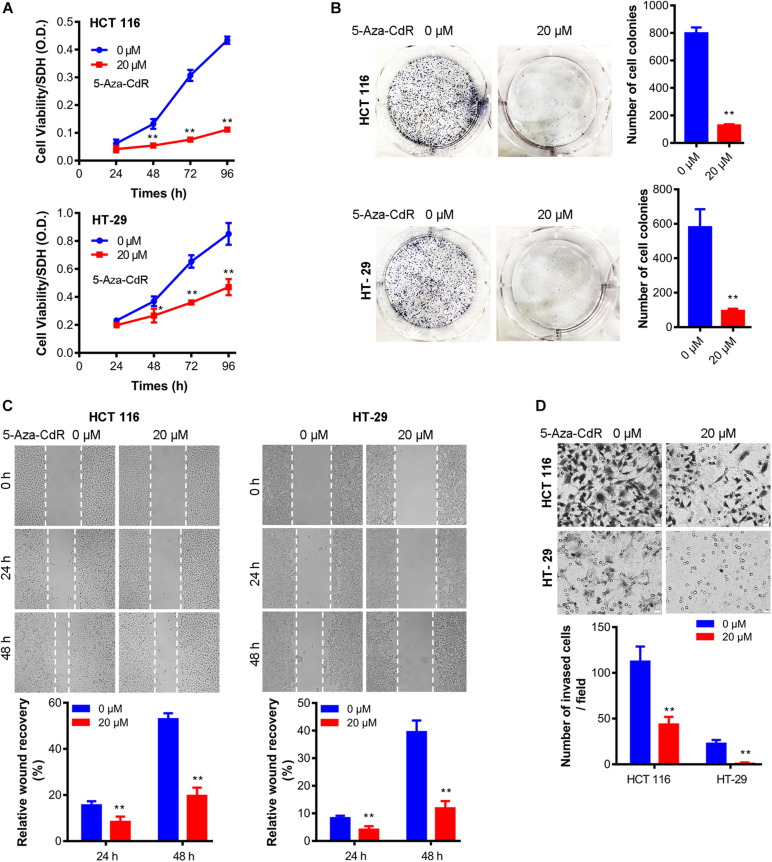
5-Aza-CdR suppressed oncogenic properties of colon cancer cells. Cell was treated with 5-Aza-CdR at indicated concentrations for indicated hours. MTT cell proliferation assay **(A)**, clonogenic growth at 14 days **(B)**, wound healing assay **(C)**, and cell invasion at 48 h **(D)** were measured. *N* = 3 independent experiments. *t*-test, *P < 0.05, **P < 0.01. Representative images were shown.

Lastly, we examined the biological effects of methylation inhibition. Results show that cell proliferation, clonegenic growth, cell migration, cell invasion were all decreased in presence of 5-Aza-CdR (Figure 4), indicating that the epigenetic suppression of CHK expression contributes to the oncogenic properties of colon cancer cells.

## Discussion

The fact that Csk was not able to fully inhibit Src kinase activity, and CHK siRNA work additively with Csk siRNA in upregulating Src activity in colon cells (Zhu et al., 2008) suggests that upregulation of CHK protein levels could collaborate with Csk upregulation in maximizing downregulating Src kinase activity. Our work in this report suggests that this could be done by epigenetically upregulating CHK transcript expression, including but not limited to downregulation of DNMTs such as employing DNMT inhibitors or decreasing DNMT protein levels.

The recent study also demonstrates interplay between CHK and Csk in hematopoietic cells (Nagy et al., 2020). The feedback upregulation of CHK expression induced by absence of Csk suggests an auxiliary role of CHK in the hematopoietic cells. Therefore, it’s possible that upregulation of CHK could reduce the drug resistance effect of cancer treatment with Csk upregulation.

Src inhibitors have been used for clinical studies, and clinical trials showed that the efficacy of several Src inhibitors in solid tumor as modest (Elias and Ditzel, 2015). The underlying reason is not known, but it is very likely the drug resistance mechanism(s) play important roles in this regard. It is possible that epigenetic CHK upregulation could also help in overcoming the drug resistance of Src kinase inhibitors, especially considering the fact that CHK inhibits Src activity mainly not *via* phosphorylating Src. Furthermore, the fact that CHK inhibits members of Src family kinases (SFKs) may also help overcome the potential drug resistance due to the feedback upregulation of other members of SFKs induced by specific Src inhibitor(s). Therefore, although novel Src inhibitors are still being developed (Yang et al., 2019; Weng et al., 2020), the role of CHK in cancer treatment with Src inhibitor(s) should be considered.

Levels of DNMT proteins were found upregulated in colon cancer cells in our study. It has been reported that knocking down of DNMT1 in colon cancer cells resulted in global demethylation of CpG sites, suggesting that DNMT1 is important for global methylation of CpG sites (Robert et al., 2003). Therefore, DNMT1 is likely important for the methylation of CpG sites of CHK in colon cancer cells we reported. Meanwhile, knocking down of DNMT1 in colon cancer cells significantly increased the ability of 5-Aza-CdR to increase expression of CDKN2A, indicating that DNMT1 may work additively or synergistically with other members of DNMT in regulating DNA methylation (Robert et al., 2003). These results are in line with our observation of the elevated levels of multiple members of DNMT in colon cancer cells, and suggest that the elevation of multiple members of DNMT may cooperate to increase the methylation of CHK gene, leading to the downregulation of CHK expression in the cells. It would also be interesting to find out the underlying mechanism of DNMT upregulation. This mechanism may also be a plausible strategy in further upregulating CHK levels. On the other hand, although epigenetics appears to be involved in regulating CHK expression, other regulating mechanism should not be excluded, for example, degradation of CHK mRNA or protein.

The significant biological effects associated with the epigenetic regulation of CHK expression in colon cancer cells including cell proliferation, wound healing, and cell invasion, support further exploration of the molecular roles and regulation of CHK.

## Data Availability Statement

The original contributions presented in the study are included in the article/Supplementary Material, further inquiries can be directed to the corresponding author.

## Author Contributions

SZ conceived and designed the study. QW and YaZ conducted the experiments and performed statistical analysis with help from others. SZ, YaZ, and QW wrote the manuscript with input from all co-authors. YiZ conducted surgery for clinical samples, and contributed to manuscript preparation with XG. All authors contributed to the article and approved the submitted version.

## Conflict of Interest

The authors declare that the research was conducted in the absence of any commercial or financial relationships that could be construed as a potential conflict of interest.

## Abbreviations

CHK: Csk-homologous kinase
Csk: c-Src tyrosine kinase
Ct: cycle threshold
DNMT: DNA methyltransferase
MTT: methyl-thiazolyldiphenyl-tetrazoliumbromide
SFKs: Src family kinases.
