# Supplementary material for: CHK Methylation Is Elevated in Colon Cancer Cells and Contributes to the Oncogenic Properties
Source: Front Cell Dev Biol. 2021 Jun 29;9:708038. doi: 10.3389/fcell.2021.708038 (PMC8276677; doi:10.3389/fcell.2021.708038)
Supplement: Supplementary file 1 [file Table_1.DOCX]

Supplementary Material





**Supplementary Figure 1. DNMT protein levels were increased in colon cancer cells.** 20 μg of total protein of cell lysates of the indicated cells (A) or tissues (B) was loaded for immunoblotting. Representative result of three independent experiments were shown.
